# *Helicobacter pylori* and gastric cancer: a lysosomal protease perspective

**DOI:** 10.1007/s10120-021-01272-8

**Published:** 2021-12-16

**Authors:** Surinder M. Soond, Andrey A. Zamyatnin

**Affiliations:** 1 Department of Immunology, Faculty of Health and Medical Sciences, University of Surrey, Guildford, GU2 7XH UK; 2 Institute of Molecular Medicine, Sechenov First Moscow State Medical University, Trubetskaya str. 8-2, Moscow, 119991 Russian Federation; 3 Department of Biotechnology, Sirius University of Science and Technology, 1 Olympic Ave, Sochi, 354340 Russian Federation; 4 Belozersky Institute of Physico-Chemical Biology, Lomonosov Moscow State University, Moscow, 119992 Russian Federation

**Keywords:** *Helicobacter pylori*, Gastric cancer, Cathepsins, Inflammation, Extracellular matrix

## Abstract

The intimate involvement of pathogens with the heightened risk for developing certain cancers is an area of research that has captured a great deal of attention over the last 10 years. One firmly established paradigm that highlights this aspect of disease progression is in the instance of *Helicobacter pylori* infection and the contribution it makes in elevating the risk for developing gastric cancer. Whilst the molecular mechanisms that pinpoint the contribution that this microorganism inflicts towards host cells during gastric cancer initiation have come into greater focus, another picture that has also emerged is one that implicates the host’s immune system, and the chronic inflammation that can arise therefrom, as being a central contributory factor in disease progression. Consequently, when taken with the underlying role that the extracellular matrix plays in the development of most cancers, and how this dynamic can be modulated by proteases expressed from the tumor or inflammatory cells, a complex and detailed relationship shared between the individual cellular components and their surroundings is coming into focus. In this review article, we draw attention to the emerging role played by the cathepsin proteases in modulating the stage-specific progression of *Helicobacter pylori*-initiated gastric cancer and the underlying immune response, while highlighting the therapeutic significance of this dynamic and how it may be amenable for novel intervention strategies within a basic research or clinical setting.

## Introduction

According to 2018 World Health Organization (WHO) and Global Burden of Cancer (GLOBOCAN) estimates, gastric cancer (GC) is the third and fourth leading cause of annual cancer mortality in respective males and females, from the total estimate of 9.6 million cancer deaths worldwide [1]. Generally, GC arises from the complex interplay between a number of important factors arising from host genetics [2], the environment, lifestyle factors and microbial factors [3]. From the latter, the gut microbiome takes on great significance as it represents the largest and the most diverse microbial ecosystem in the human body and serves to generally support the host’s mucosal immune response to eliminate pathogens [4]. The importance of inflammation in response to pathogens and the specific role it plays in cancer progression had been noted as being of key importance, as reported by Rudolf Virchow as far back as 1863, where the origins of certain cancers were positively correlated with sites of persistent inflammation [5]. Subsequently, microbes or microbiota and their inflammatory effects have been increasingly recognized in as much as 20% of all cancers [6] and more specifically in colorectal cancer [7, 8], prostate cancer [9], colon cancer [10] and gallbladder cancer [11].

Of significant relevance is the Gram-negative bacterium *Helicobacter pylori* (*H. pylori*), which has been reported to asymptomatically colonize over 50% of the world’s population and can become established within the gastric lining of individuals early on in life [12]. As a likely precursor for the carcinogenesis of the stomach, it has consequently been categorized as a Class 1 carcinogen, by the WHO [13]. While 10% of *H. pylori* infections are associated with peptic ulcer disease, around 1–3% if those infected will progress to GC with an estimated survival rate of under 5 years [14]. Moreover, a further 0.1% will develop mucosa-associated lymphoid tissue (MALT) lymphoma [15]. The development of this scientific paradigm of key importance was indeed grounded upon the milestone discovery of the Gram-negative bacteria *H. pylori* as being the infectious agent that caused chronic gastritis [16–19], atrophic gastritis, intestinal metaplasia, dysplasia and carcinoma of the stomach, thus contributing to the histopathological Correa model for gastric carcinogenesis [3, 20].

Mechanistically, the virulent actions of the *H. pylori* cytotoxicity-associated gene A (CagA) and vacuolating cytotoxic A (VacA) proteins on gastric mucosa cells can instigate a complex array of biological effects ranging from the production of *pro*-inflammatory cytokines and the recruitment of immune cells to the site of infection, to triggering gastric epithelial cell survival responses [15, 20]. To ensure its survival and disease progression, *H. pylori* can suppress the activities of phagocytic cells and T-cell functions during infection [21], while catalyzing the formation of urea as a way of assuring its own survival under the harsh low pH conditions of the stomach [21]. Additionally, the by-products of *H. pylori* metabolism can be significantly destructive to the host’s epithelial cells and contribute to the carcinogenic effects of *H. pylori* infection.

At the cellular and molecular levels, of central importance is the extracellular matrix (ECM) and the dynamic nature of which permits tumor-associated inflammatory cells to become established, while also directly contributing to angiogenesis of the tumor and the mobilization of tumor cells [22–24]. Central to ECM modulation are the matrix metalloproteases (MMPs) and the cathepsin proteases, which over the last 10 years have gained importance based on the contribution of their expression during tumor development and metastasis [25–27]. Consequently, of importance is the question of how such proteases can holistically modulate the ECM, modulate the inflammatory- and adaptive-immune responses directed by neutrophils, macrophages, dendritic cells or cytotoxic T cells (CTLs), and to favor tumor cell growth [28]. Collectively, as central regulators of a complex series of biological effects that are central to GC progression, the cathepsin proteases, do have exceptional potential to be therapeutically targeted, with a number of agents currently being evaluated for therapeutic purposes [29, 30].

**Fig. 1:**
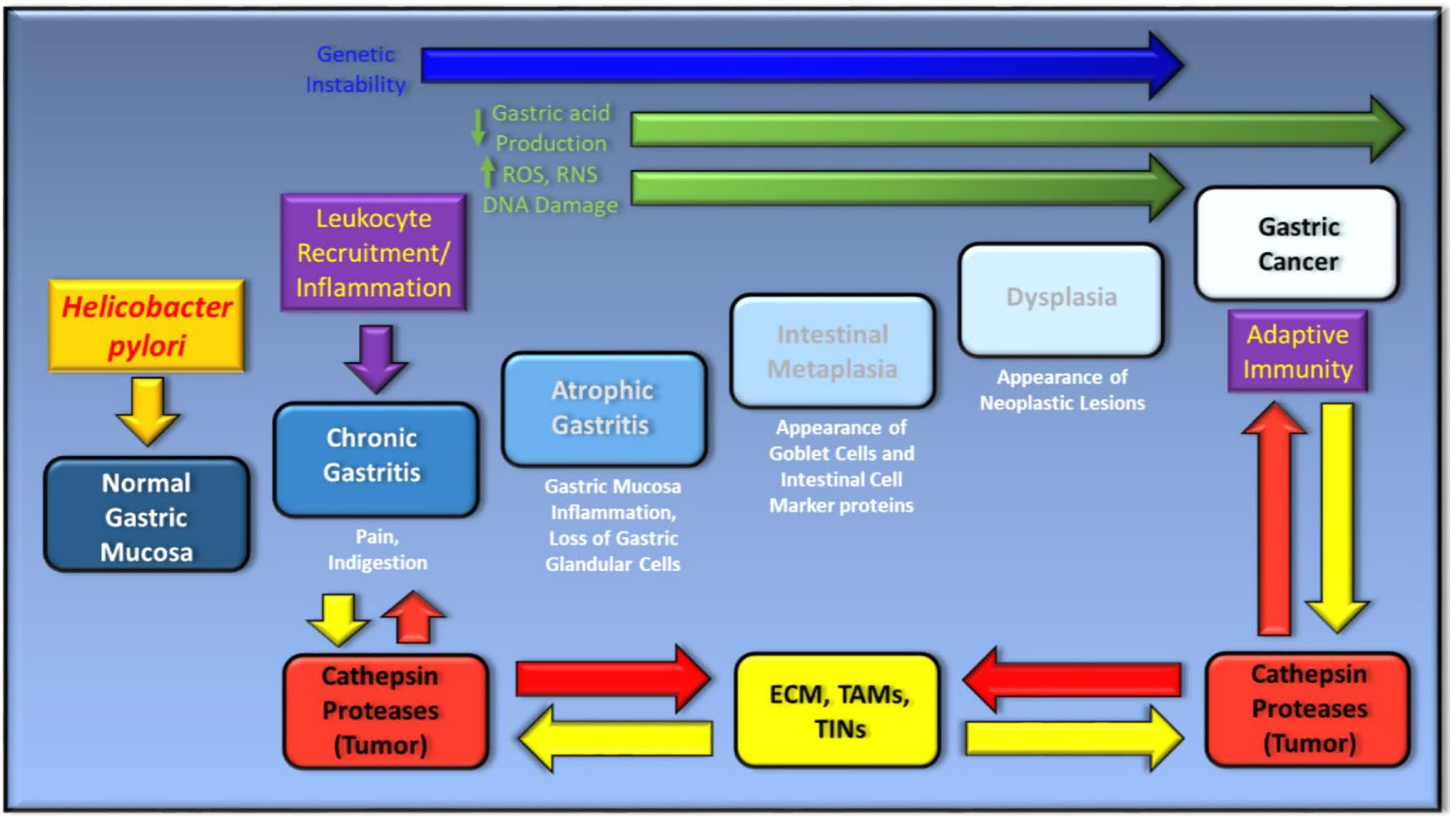
*Helicobacter pylori* infection and key contributing factors in the progression of gastric cancer. *H. Pylori infection* of the gastric mucosa induces chronic inflammation (orange arrow), which can drive the development of gastric cancer through the interplay of genetic factors (blue arrows), key biochemical factors (green arrows) and extracellular or cellular factors from the immune system (yellow arrows). The cathepsin proteases (red boxes) and their regulatory input into modulating the extracellular matrix (ECM), tumor-associated macrophages (TAMs), tumor-infiltrating neutrophils (TINs), the innate and adaptive immune response are highlighted (red arrows). Tumor-derived cathepsin proteases can also be modulated by components derived from the ECM, TAMs, TINs and the innate or adaptive immune response (yellow arrows)

In this article, we focus on the relationship that has developed between *H. pylori* and GC progression, through building on the importance of pathogen-induced inflammation and the immune response as a central theme, whilst detailing additional emerging molecular mechanisms of significance that are responsible for this relationship to take effect, namely through the actions of the cathepsin proteases toward the ECM (Fig. 1). By unveiling the interplay of these molecular mechanisms, we identify key axes of regulation, which may hold potential in being therapeutically targeted, either at the basic research level or within the clinic.

## *Helicobacter pylori* infection and gastric cancer progression

**Fig. 2:**
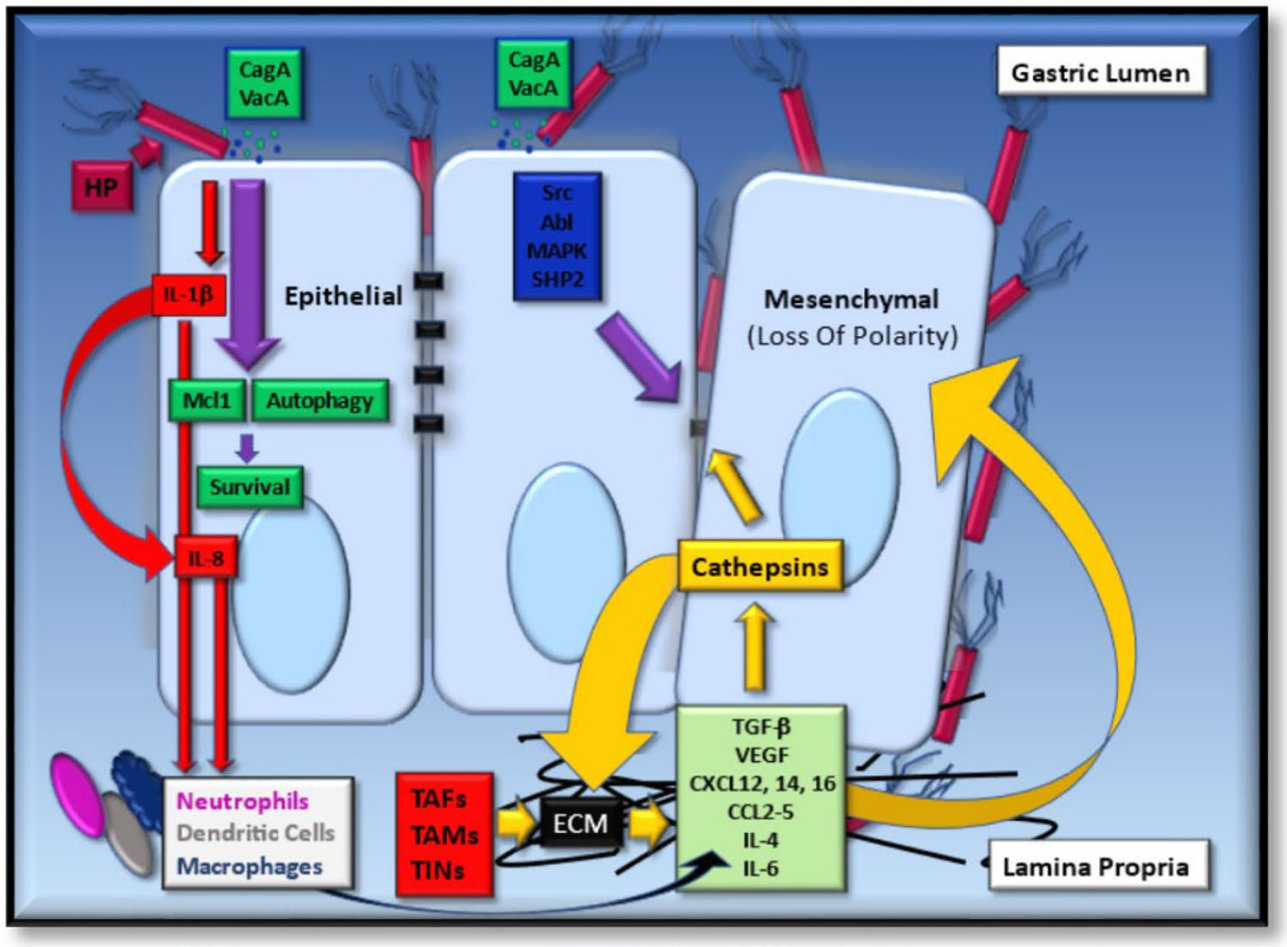
The localized inflammatory response upon the infection of gastric epithelial cells by *H. pylori* (HP). Gastric epithelial responses to HP infection through the actions of CagA and VacA result in activation of cell survival through Mcl-1 protein induction and autophagy regulation. IL1β and IL-8 expression (red arrows) is also upregulated which induce the influx of inflammatory cells. Persistent inflammation upregulates the expression of a number of cytokines and chemokines (pale green box), which can contribute to epithelial gastric cancer cells differentiating to mesenchymal cells. Such signaling cues can also enhance the expression and secretion of cathepsin proteases (yellow box and arrows), which can act on modifying the extracellular matrix (ECM, black box), along with cathepsins derived from tumor-associated fibroblasts (TAFs), -macrophages (TAMs) and tumor-infiltrating neutrophils (TINs). As contributing mechanisms to gastric cancer progression, cathepsins can also target the cleavage of cell–cell junctions (black tabs), the expression of which can also be negatively modulated upon the activation of signal transduction pathway intermediates by CagA and VacA (blue box)

*Helicobacter pylori* infection can change the composition of the gastric microbiota by raising the pH, allowing the creation of a niche for bacterial colonization, and which favors consistent and prolonged *H. pylori* infection. Consequently, gastritis, gastric ulcer, atrophy and gastric cancer can progressively develop, as depicted in the proposed Correa model for GC development in Fig. 1 [20]. Therapeutically, the eradication of *H. pylori* can prevent the onset of GC as seen from treating infected patients suffering from *H. pylori*-induced gastritis [31] and who lack pre-cancerous lesions, culminating in decreased incidents of GC [32]. However, in dyspepsia patients classified using Operative Link for Gastritis Assessment (OLGA) staging, the eradication of *H. pylori* in stages III–IV does not appear to abolish the risk for neoplastic progression [33]. Collectively, such observations support the belief that *H. pylori* may play a role in the early stages of GC development and that during the stages of atrophy and metaplasia that follow, there could be other contributing mechanisms of importance. Nevertheless, it has become firmly established that inflammation is a key contributing factor to these defined stages of GC development [28]. More specifically, and during chronic gastritis, there is an influx of macrophages, neutrophils, and plasma-derived bone marrow-derived stem cells (BMDSC), which are a source of *pro*-inflammatory cytokines, reactive oxygen (ROS) and nitrogen (RNS) species. At the molecular level, this response is instigated by the *H. pylori*-derived CagA and VacA virulence factors through the pathogens T4SS system [34–36]. Such factors are genetically encoded by the bacterial *Cag* pathogenicity island (CagPAI) [34], and is an essential locus that contributes to the formation of pre-cancerous lesions in the host [37]. Approximately, 100% of East Asian and 70% of Western *H. pylori* strains encode the *CagA* gene [34, 38], which along with the VacA protein can be shed from the bacteria in response to bacterial stress in the form of bacterial outer membrane vesicles (OMVs)[39, 40]. Once inside the cell, the CagA protein can undergo phosphorylation by Src and Abl kinases [41, 42] and activate the SHP2 phosphatase, the interplay of which can collectively contribute to cellular morphological changes, such as enhanced cellular elongation and cell scattering [35, 42, 43]. Here, mitogen-activated protein kinase activation (MAPK) is a contributing factor [38], as is the modulation of epithelial cell junction integrity, which alters epithelial permeability and cellular polarity [38]. Moreover, through the activation of MAPKs and anti-apoptotic MCL1 protein expression, intracellular CagA can counteract VacA-mediated stress-induced apoptosis of mammalian cells [44], through dampening VacA-enhanced endoplasmic reticulum (ER) stress-induced C/EBP homologous transcription factor protein (CHOP) expression (Fig. 2) [45]. An additional and alternative mechanism proposed for VacA-mediated apoptosis regulation occurs through the modulation of mitochondrial outer membrane permeabilization (MOMP), and its activation of the intrinsic arm of the apoptosis pathway [46]. VacA can also induce autophagy and CagA degradation through ROS expression and Akt kinase activation [47]. However, persistent exposure of epithelial cells to VacA can deregulate autophagy through disarming autophagosomes, promoting cell survival and accumulating ROS expression, thus potentiating localized inflammation and cellular carcinogenesis [48, 49].

## *Helicobacter pylori*, inflammation and the immune response

The chronic gastritis phase of *H. pylori* infection takes on additional seriousness from the perspective of recruiting immune cells, which are a significant source of pro-inflammatory cytokines or ROS and RNS species, with very distinct mechanistic effects during GC development. Initially, neutrophils are directed to the gastric mucosa in response to the actions of CagA-mediated activation of transcription factor NF-κB, which can induce IL-1β expression and secretion from gastric epithelial cells [50, 51]. Subsequently, macrophages and dendritic cells are recruited as a consequence of enhanced permeability of the gastric mucosa [52, 53]. Contributing to this effect is the IL-1β-dependent expression of IL-8, which, through a positive feedback loop and drives enhanced gastric IL-1β expression, culminating in enhanced mucosal inflammation [54] and low gastric acid output, which are necessary precursor steps for gastric atrophy [55], hyperplasia and GC [20], even in the absence of an adaptive immune system [56]. Here, of specific importance are the pathogen recognition receptors, such as the Toll-like receptors (TLRs) and nucleotide-binding oligomerization domain (NOD)-like receptors (NLRs) that are found on the surface of epithelial, macrophage, neutrophil and dendritic cells [57]. Their occupation, by unique bacterial pathogen-associated molecular pattern proteins (PAMPs), can generally signal through the MyD88 and TRIF protein intermediates that activate NF-κB, AP-1 and IRF3 [58], which mediate the upregulated expression of the cytokines IL-8, TNF-α, IL-1β, IL-6, IL-10 and IFN-γ [59]. Moreover, NLRs and leucine-rich repeat-containing proteins accessorize TLR signaling to form the inflammasome [60], which is fundamental to IL-β and IL-18 cytokine maturation. Briefly, NF-κB activation can upregulate *pro*-IL-1β, *pro*-IL-18 and NLRP3 protein expression, the latter of which permits the recruitment of *pro*-caspase-1 using the adaptor protein ASC. Through this PAMP/TLR/NLR complex, caspase-1 can become activated and cleaves IL-1β and IL-18 precursors giving rise to their mature forms, which are secreted in a gasdermin-d-dependent manner [61]. NRLP3 inflammasome-containing cells can subsequently die by pyroptosis, thus restricting the growth of intracellular bacteria [61]. Interestingly, while this may be one way in which the mucosal epithelial layer is breached, another is by the epigenetic loss of e-cadherin expression, through the methylation of its gene promoter, as seen in the presence of IL-1β and CagA stimulation of cancer cells (Fig. 2) [62, 63].

From an immunity perspective, a key mammalian defense mechanism for bacterial clearance is phagocytosis. *H. pylori* encodes the sialic acid-binding adhesion (SabA) and blood group antigen-binding adhesion (BabA) proteins, which permit *H. pylori* binding to epithelial cells [64]. SabA can also bind human neutrophil receptors, and in doing so induce phagocytosis and ROS production [34]. Following phagocytosis, phagosomes may also fail to mature through the activity of VacA, and instead resemble early endosomes that exhibit deficient lysosome fusion abilities particularly for highly virulent type I strains of *H. pylori* infection in professional APCs [65]. Nevertheless, following phagocytosis, *H. pylori* can express arginase 2, which reduces NO or O_2_^−^ radicals and contributes to *H. pylori* survival [66, 67]. This can also be achieved through the bacterial expression of SOD, catalase and peroxiredoxins [67]. Collectively, such mechanisms contribute to *H. pylori* survival. The subsequent formation of ROS can activate caspase-1, thus enhancing inflammasome activity and pro-inflammatory cytokine secretion [68].

In summary, epithelial cells can respond to *H. pylori* infections upon TLR receptor occupation, while inflowing immune cells respond by TLR occupation and phagocytosis. The production and secretion of *pro*-inflammatory cytokines mediate inflammation during chronic gastritis and atrophic gastritis during GC progression, for which the ECM is a central regulator (Fig. 2).

## The extracellular matrix and its contribution to gastric cancer progression

Over the years, the ECM has received particular attention as a key regulator for tumor progression from the perspective of how its plasticity can alter the growth and metastatic potential of tumors and their response to biochemical cues originating from associated stromal cells [69]. In light of it maintaining essential aspects of tissue architecture and the organization of epithelial and auxiliary cells, the most important aspects of the ECM include its ability to modulate cellular survival, growth, and differentiation [70], morphogenesis, cellular adhesion, polarity and migration [71]. In the context of gastric epithelial cells and GC, how the ECM modulates the tumor microenvironment (TME) has revealed a number of very important findings, related to how pre-malignant lesions progress toward gastric adenocarcinoma in the presence of inflammation [72]. This process can be largely regulated through alterations in the rate of the synthesis and breakdown of structural and cellular components of the ECM [70].

Of particular relevance here are the stromal cells, some of which originate from bone marrow-derived stem cells (BMDSC) that can be recruited to chronically inflamed tissues [73] in response to IL-1β, IL-6, TNF-α and the chemokine CXCL12 [74, 75]. At the tumor site, BMDSCs also have the capacity to differentiate into angiogenesis-promoting endothelial cells [76, 77], or TAFs [78] as a source of extracellular protease expression, TGF-β, VEGF, CXCL-12, -14, -16, CCL2-5, IL-4 or IL-6 [79, 80], the collective interplay of which contributes to tumor progression. For example, TAF-derived IL-6 expression can direct the differentiation of monocytes to macrophages [81], while TAFs can also contribute to polarization of M1 (*pro*-inflammatory) macrophages to the M2 (*anti*-inflammatory) phenotype [82] and modulate immunosuppression through TGF-β expression [83]. Here, macrophages can constitute over 50% of the tumor mass [84, 85], from which the M2 sub-type can support tumor survival and proliferation [86]. Similarly, tumor-infiltrating neutrophils (TINs) can help develop an immune-suppressive TME, through cytokine or chemokine release [87–90], while also having the capacity to differentiate into an N2 phenotype [91]. In the context of GC progression [92], TINs are believed to promote this by enhancing EMT of the gastric epithelium, through the activation of the ERK and JAK/STAT signaling pathways [93] or in response to IL-17 (Fig. 2) [94–96].

## Cathepsin proteases as key regulators of the extracellular matrix and tumor microenvironment

**Fig. 3:**
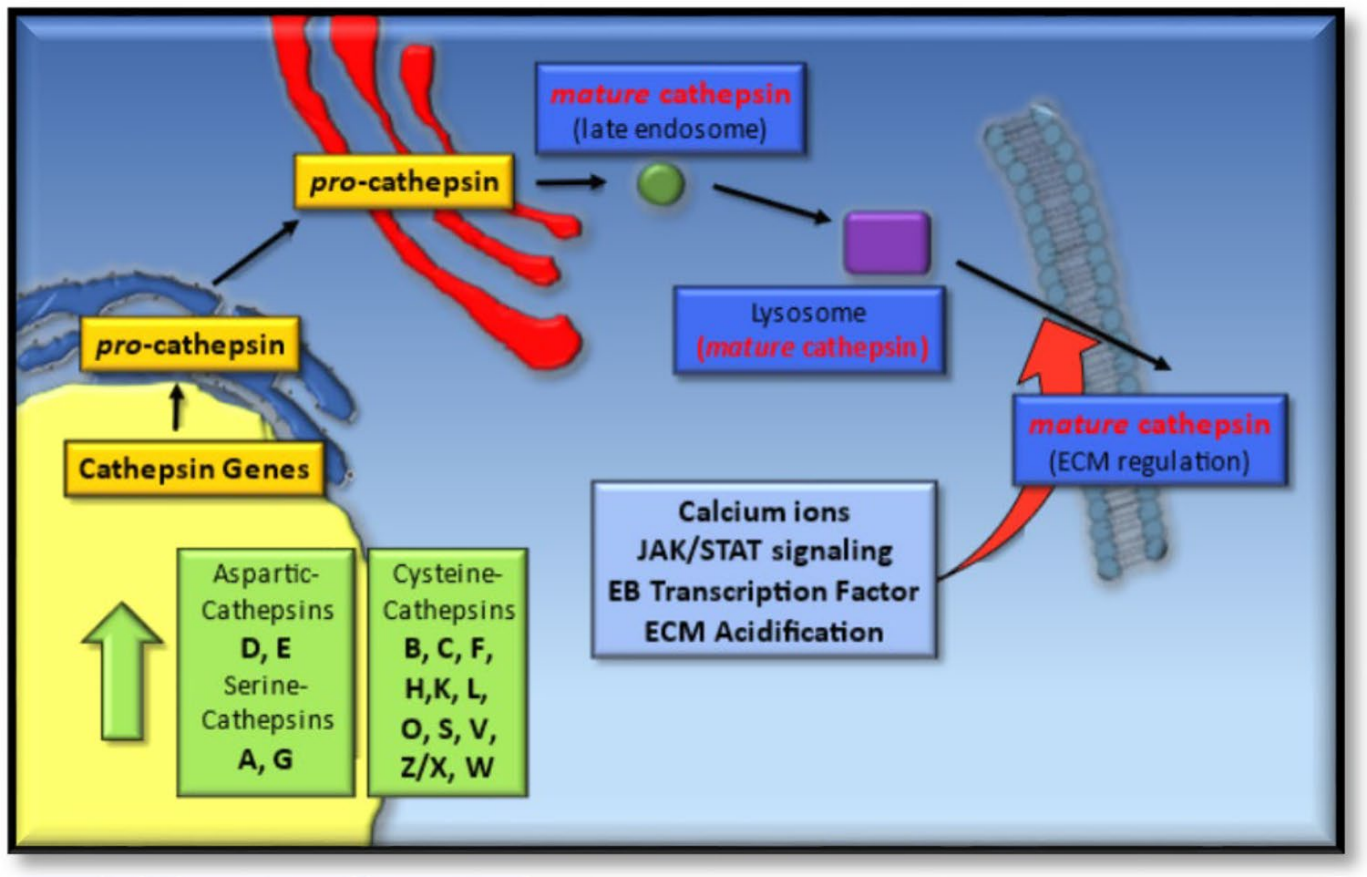
Cathepsin gene expression and protein trafficking in cancer and stromal cells. All 15 cathepsin proteases are synthesized as catalytically inactive pro-cathepsins, which are glycosylated as they pass through the secretory pathway. Maturation of pro-cathepsin proteases occurs upon their trafficking through the endosome or lysosome, resulting in their pro-domain cleavage and removal. Under conditions of enhanced gene expression or specific cell-stimulatory cues, some cathepsin proteases are secreted and modulate the extracellular matrix (ECM)

A rapidly emerging group of proteases that can modulate the TME through the proteolysis of ECM components [97], membrane-associated cytokines [98], receptor proteins [99, 100] or cell–cell junction proteins, are the cathepsin proteases [101]. While originally characterized as important enzymes for lysosomal function, the cathepsins have recently come in to focus as being important in regulating a number of key aspects of disease pathology. Briefly, over 15 cathepsin proteases have been identified and can be sub-grouped into aspartic-, serine- and cysteine proteases, which are post-translationally processed as they traverse the secretory pathway and come to reside in the lysosomes [27]. However, under certain conditions (as in a number of cancer types), cathepsin protease expression is enhanced and significant levels of specific cathepsins are secreted to the extracellular milieu [102–105], either as mature proteases or inactive zymogens that can become activated during a decrease in the extracellular pH, Fig. 3 [106, 107].

Their extracellular localization is more common in a number of pathological conditions such as cancer, and can be derived from a number of sources, such as tumor cells or the auxiliary cells from the stroma [103, 104]. Mechanistically, secretion of cathepsins is thought to occur by vesicular exocytosis through lysosomes fusing with the plasma membrane, which can be triggered through increased intracellular Ca^2+^ ions [126, 127], JAK/STAT signaling [128, 129], or transcription factor EB activation [130]. Acidification of the ECM is another biochemical cue that enhances cathepsin secretion and is predominant within the microenvironment of tumors [131], atherosclerosis [132] and osteoarthritis [130]. Alternatively, increased levels of intracellular ROS or tumor suppressor protein p53 can also enhance leakage of some cathepsins through lysosomal membrane permeabilization (LMP), giving rise to their cytoplasmic localization [27, 133, 134]. Cathepsin proteases secreted into the ECM upon their overexpression, which have been diagnostically associated with the progression of certain cancer types or patient prognosis, are highlighted in Table 1 [29, 30].

**Table 1:** Cathepsin proteases reported to be overexpressed by specific cancers

Cathepsin	Amino acids (kDa)	Cancer type	References
A	480 (54)	MM	[108, 109]
B	339 (38)	MM, L, B, C, GB, H	[110, 111]
D	412 (45)	B, L, O	[112, 113]
H	335 (41)	GB	[114, 115]
K	329 (39)	SCC, BCC, G	[116, 117]
L	333 (38)	PC	[118–120]
S	331 (37)	C, GC	[121, 122]
Z/X	303 (34)	PC, H	[123–125]

The amino acids and sizes of the cathepsin proteases overexpressed in malignant melanoma (MM), lung (L), breast (B), colon (C), glioblastoma (GB), hepatocarcinoma (H), ovarian (O), squamous cell carcinoma (SCC), basal cell carcinoma (BCC), glioma (G), prostate cancer (PC) and gastric cancer (GC) are highlighted

Of central regulatory importance is the auto-catalytic activation of cysteine cathepsins (B, C, F, H, K, L, O, S, V, Z/X, W) under mildly reducing and acidic conditions [135, 136], and which at neutral pH can be activated by negatively charged glycosaminoglycans (GAG) such as keratin and chondroitin sulfates [137], as seen for cathepsin K regulation [138]. For some of these cysteine cathepsins, their extracellular localization can give rise to a loss of activity, due to the neutral pH of the microenvironment [135]. However, such conditions can offer some favorable outcomes for other cysteine cathepsins through their stabilization, particularly if these proteases are secreted in their inactive zymogenic forms, and which can become auto-activated when a favorable drop in extracellular pH occurs through extracellular acidification. Such a pH-dependent auto-activation mechanism has been reported for pro-cathepsins B, K, L and S and their ability to cleave thyroglobin, in vitro [106]. Secreted mature cathepsins have the capacity to degrade ECM components [102], some of which can also modulate ECM signaling through their digestion products acting in an autocrine (or paracrine) manner (Table 2) and enhancing cathepsin expression or secretion [139].

**Table 2:** Cathepsin protease expression, their extracellular target proteins and the biological effects of their expression during cancer progression

Cathepsin	ECM substrate	Biological effects	References
B, L, S	E-cadherin	Tumor invasiveness	[140]
B	CD18	Angiogenesis	[100]
S	Nidogen-1	NSLC invasiveness/angiogenesis	[141]
S	Canstaten/arresten	Angiogenesis	[142]
B, L	Lam./Fibron./COL IV	Tumor invasiveness	[143]
B	Tenascin-C	Oncogenesis	[144]
B, L, S	COL XVIII	Angiogenesis	[145, 146]
K	Periostin	Breast cancer metastases	[147]
K	SPARC	Bone metastases	[148]
K,S,V	Elastin	Cardiovascular disease	[149]
B,L	Perlecan	Neuroprotection	[150]

*Lam.* laminin, *Fibron.* fibronectin, *COL* collagen, *SPARC* secreted protein acidic and rich in cysteine protein, *NSLC* non-small lung cancer

Collectively, such regulatory aspects of cathepsin proteases have a high level of significance in them being able contribute to a number of important aspects of cancer pathology, such as the modulation of the ECM and infiltration of inflammatory cells and tumor development, growth and dispersal under extreme physiological conditions. As the mechanistic insights of how cathepsins are regulated transcriptionally and at the protein level may be cell type and context dependent, greater focus is indeed needed in delineating the effects of these factors exclusively in gastric cancer cell systems. Nevertheless, based on their significant input toward general cancer progression, they have long been viewed as good candidates for therapeutic targeting in addition to potential diagnostic or prognosis markers [29].

## Tumor and immune cell regulation by extracellular cathepsin proteases

In addition to cathepsins secretion being enhanced from tumor cells, such proteases are also secreted from immune cells during inflammation (particularly from macrophages), thus directly regulating ECM dynamics and immune cell influx [98, 99]. In addition to cleaving components of the ECM such as collagen and elastin [138, 151–154], cathepsins can also ‘shed’ inflammatory cytokines, chemokines [155–159] or signaling receptor proteins [99, 152, 160], all of which have established or emerging importance in the co-modulation of inflammation and tumor progression. Of significance here are cathepsins K, A, G and E, which have been demonstrated to be constitutively expressed within the epithelium of gastrointestinal cells of the stomach [27], with cathepsin D expression being recognized as a potentially reliably independent prognostic marker for GC [161]. Moreover, cathepsin S overexpression has been associated with GC invasiveness [162], and as a possible diagnostic and prognostic marker [163] for inflammation-induced spasmolytic polypeptide/trefoil factor 2-expressing metaplasia (SPEM) [164]. While some cathepsins have a limited range of tissue expression basally, it must be noted that some cathepsins, which are otherwise expressed at low levels, can be inducibly expressed and secreted under specific physiologically relevant conditions. This has been reported for enhanced cathepsin S expression under IL-1α and TNF-α stimulatory conditions in human chondrocytes [128], and for cathepsin K expression under RANKL-stimulatory conditions in human osteoclast cells [165]. Moreover, some cathepsins have also taken on some importance based on potential changes in their expression profiles throughout the various stages leading to GC, the significance of which is coming into focus, as a broader picture develops from changes in their molecular expression to biological effects and disease progression (Table 3).

**Table 3:** The stage-specific effects of intracellular cathepsin protease expression during gastric cancer development

Stage	Cathepsins (tissues)	Effects	References
Chronic gastritis	−X (+) (GM, MAC, AM),	Invasiveness	[115]
	−Z (−) (GM)	EP, MI	[166]
	−B, −L, −K (nc) (E, GM)	Late-stage GC	[167]
	−X (+) (E)	Shedding	[167]
	−W (+) (NK, CTLs)	Cytotoxicity	[167]
Atrophic gastritis	−W (+) (NK, CTLs)	Cytotoxicity	[168]
	−Z (−) (GM)	EP, MI	[166]
	−B (+) (E, GM)	GCD	[169]
	−L (+) (E, GM)	GCD	[169]
Intestinal metaplasia	−Z (−) (GM)	EP, MI	[166]
	−B (+) (E, GM)	GCD	[169]
	−L (+) (E, GM)	GCD	[169]
	−E (−) (GM)	Dedifferentiation	[170]
	−E (+) (GM, IMG)	–	[171]
Dysplasia	−B (+) (E, GM)	GCD	[169]
	−L (+) (E, GM)	GCD	[169]
	−E (−) (GM)	Dedifferentiation	[170]
Gastric cancer	−X (+) (GCAR)	Invasiveness	[115]
	−B (+) (E)	Invasiveness	[172]
	−B (+)/−L (+) (GM)	Proliferation	[173, 174]

Cell-specific cathepsin proteases expression profiles are highlighted in relation to the biological effects they have been linked to during GC development (GCD). *GM* gastric mucosa, *MAC* macrophage, *AM* antral mucosa, *E* epithelial, *EP* epithelial proliferation, *MI* macrophage infiltration, *NK* natural killer cells, *CTL* cytotoxic T cells, *IMG* intestinal metaplastic glands, *GCAR* gastric carcinoma cells, *CD* cellular differentiation. Expression patterns are highlighted as induced (+), deficiency (−) and no change (nc)

Additionally, infiltrating macrophages (cathepsins F, K, O), cytotoxic T lymphocytes (cathepsins C, W), APC (cathepsin S), monocytes (cathepsin G) and neutrophils (cathepsin G) also express specific cathepsin proteases. Here, cathepsin B is of importance based upon mouse myeloid-derived suppressor cells (MDSCs) failing to accumulate in the absence of cathepsin B expression, possibly due to the lack of TNF-α-derived signaling cues [175, 176], whereas cathepsin K can contribute to macrophage infiltration and upon overexpression of cathepsin B can give elevated CCL2 and COX2 expression [177], thus favoring MDSC expansion. Additionally, CCL2 has been reported as potently attracting BMDSCs, such as TAMs, to the tumor site [178] and have the capacity to upregulate cathepsin expression based on their responsiveness to IL-4, -6 and -10 stimulation [98]. The significance of TAMs and the effects of their cathepsin expression capabilities in disease progression have been highlighted by a number of excellent mouse studies [179]. For example, TAM-derived cathepsins B, H and S had a predominantly negative effect in RIP1-Tag2 pancreatic neuroendocrine cancer progression [98], whereas tumor-derived cathepsin L contributed to cancer progression.

The specific substrates that these cathepsins manifest their activity toward is also an emerging area of interest, which has yielded a number of insightful findings into GC progression. For example, differentiation of BMDSCs to macrophages, neutrophils and dendritic cells is driven by tumor- and ECM-derived growth factors and cytokines [180]. In support, cathepsin expression is required for macrophage survival, which is otherwise deficient during impaired autophagy responsiveness, and leads to their apoptosis through mitochondrial damage-mediated ROS generation [181]. Moreover, cathepsin S expression also contributes to the polarization of macrophages from types M1 to M2 [182], in addition to autophagosome–lysosome fusion [182], as does cathepsin K expression through its activation of TLR-4 [183], thus collectively contributing to the development of a TME that is supportive of tumor growth [184, 185]. Of note, cathepsin-mediated TLR cleavage and activation is also essential for dendritic cell function [186] as another significant cellular contributor to TME, based on TLR-4 expression synergizing with cancer cell progression by it positively regulating EMT [187].

Similarly, tumor-infiltrating neutrophils (TINs) have also been reported to shift their phenotype from N1 to the tumor-promoting N2 sub-type in a cathepsin expression-dependent manner. Here, cathepsin C plays a central role as seen in Papillon–Lefèvre syndrome in humans, where a lack of cathepsin C expression impairs neutrophil function, giving rise to erythematous palmoplantar hyperkeratosis and early-onset periodontitis [188]. Moreover, neutrophil survival has been reported to be lysosomal cathepsins B and D expression dependent, both of which can contribute to the initiation of apoptotic cascades through caspase-8 activation and BID cleavage [189, 190]. Alternatively, cathepsin D can mediate VacA proteolysis in autophagosomes and is a protease that is also required for the correct execution of autophagy [48].

As detailed, extracellular cathepsins affect immune cell physiology, through cytokine, chemokine and receptor shedding or cleavage [103]. Functionally, they can activate pro-inflammatory ELR chemokines, deactivate non-ELR chemokines or regulate chemotaxis and angiogenesis, as seen with cathepsins S, L and K [103]. Cathepsin S expression can also permit the infiltration of monocytes and macrophages through the basement membrane [191], whereas cathepsin K expression has *anti*-microbial effects in intestinal goblet cells, as seen from cathepsin K^−/−^ mice, which developed severe colitis and altered gut microbial communities [192].

**Fig. 4:**
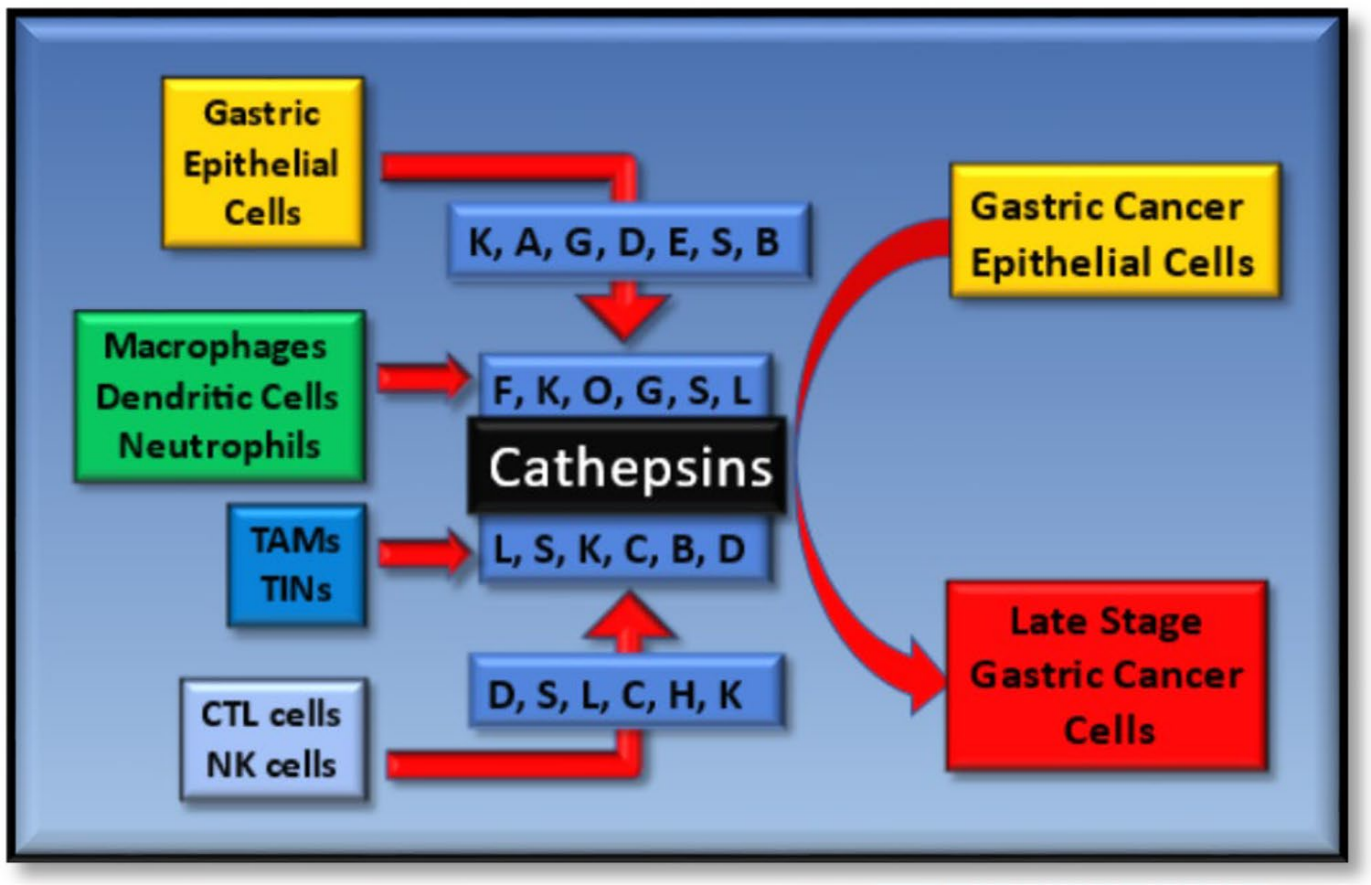
The diverse actions and cellular origins of the cathepsin proteases during gastric cancer progression. Schematic highlighting the various cathepsin proteases which have been characterized as being important in gastric cancer progression, when either derived from gastric epithelial cells or tumor-associated macrophages (TAMs), tumor-infiltrating neutrophils (TINs), Cytotoxic T cells (CTL) and natural killer cells (NK) from the innate or adaptive immune systems

In summary, tumor or immune cell-derived cathepsin proteases carry significance in modulating the TME exclusively or in concert with other ECM-derived protein factors, which can favor unbridled inflammation, tumor growth and development in a variety of ways. Such emerging regulatory relationships highlight an elevated level of complexity, based on whether the cathepsins are derived from the tumor or immune cells and whether they exhibit effects at the intracellular and extracellular levels, or a combination of both (Figs. 2, 4). In large, such findings are modeled on a diverse number of cancer-type paradigms. To clarify, research efforts are being helped through addressing the dispensability of individual cathepsin proteases, exclusively or in combinations, during cancer initiation and progression through the use of knockout-mouse studies, the findings from which could be extended to offer greater correlative insights when exclusively profiled with the various stages of the Correa model for GC development.

## Adaptive immunity regulation by intracellular cathepsin proteases

Of additional significance are the effects of cathepsin proteases in the regulation of adaptive immunity and immune surveillance, through regulating the responsiveness of natural killer (NK) cells and cytotoxic T cells (CTLs) during disease progression. Herein, while the mechanistic recognition of newly formed antigens can be recognized by CD8^+^ and CD3^+^ CTLs, through MHCI and TCR engagement, one key regulatory step for T-cell activation is the co-stimulatory secondary signal derived from CD28-CD80/CD86 engagement between T cell and antigen-presenting cell (APC) [193]. In this context, co-inhibitory signals may also arise as in the case of tumor cells expressing receptors PD-1, LAG-3, and Tim-3, which when bound to their cognate ligands on T cells, induce T-cell inactivation. In the instance of PD-1, it can bind one of its two ligands, namely PD-L1 [194–196]. Of equal importance are the MHCII molecules, which also display tumor or pathogen-derived antigens, as on APCs such as dendritic cells, or macrophages, and which subsequently bind the LAG-3 ligand on activated T cells and NK cells, and inhibit their activation [197, 198]. One alternative mechanism by which tumor cells may overcome cytotoxic CTL-mediated tumor cell death is through downregulation of tumor antigen expression, leading to a reduction in CD4^+^ T-helper cell activation and active CTLs [28]. In this context, the fate of the invariant chain ‘Li’ protein (CD74) is of fundamental importance. Specifically, it is required for MHCII-antigenic peptide charging [199], following its cleavage by the proteolytic activity of cathepsin L. However, complete deactivation of ‘Li’ protein can also occur through its degradation by other cathepsin proteases [200], such as cathepsins B [201], G [202] or S [200], thus highlighting a central immunosuppressive property of such proteases on naïve T-cell activation. Key negative regulation of MHCII protein expression can also occur under IL-6 and IL-10 stimulatory conditions and offer dendritic cell tolerance, where IL-6 signals can exclusively reduce the protein levels of the invariant chain, through enhanced cathepsin S activity [203]. Moreover, CCL2 expression has also been associated with cathepsin S-mediated cleavage of the invariant chain, after which the liberated intracellular domain of CD74 can translocate to the nucleus and activate transcription factor NF-κB and CCL2 transcription [178]. Such a mechanism could also be viewed as a feedback loop that helps drive the influx of BMDSC and TAMs to the site of the tumor.

The role of cathepsin proteases and their expression are also coming into focus as being key players in additional aspects of the inflammatory and immune response. Expression of cathepsins D or S and cystatin C were reported to modulate the differentiation of immunogenic dendritic cells to an immunotolerant phenotype in a time-dependent manner [204]. Here, TLRs have been reported to enhance immunotolerance through their regulated expression of IL-1β and IL-6 in tumor and stromal cells [205]. As demonstrated, TLRs 3, 7, and 9 can be processed by cathepsin proteases during the second stage of a two-step cleavage reaction in macrophages, dendritic cells and fibroblasts, to permit efficient TLR-nucleic acid sensing [186]. In the instance of secreted cathepsin K, it could also polarize M1 macrophages to M2 through TLR-4 processing and activation [183], which is viewed as an important aspect of M2 macrophage–tumor cross talk for EMT progression of cancer cells [187].

In combination with the above, NK cells can target poorly differentiated tumors and cancer stem cells expressing low levels of MHCI [206], through activating tumor death receptor signaling pathways or upon perforin and granzymes release. The latter can be stored as inactive precursors, which can also be cleaved and activated by cathepsin proteases L, C and H [207, 208]. Such effects can also be negatively regulated upon intracellular cystatin F expression [209], or upon the exposure of NK cells to exogenous cystatin F protein [210], which gives rise to reduced NK cell-mediated cytotoxicity [209, 211].

In summary, intracellular cathepsins D, S, L, C, H and K clearly have significant and diverse positive and negative regulatory roles within the adaptive immune response and their expression contributes to immunosurveillance, macrophage polarization, cell-mediated cytotoxicity and dendritic cell maturation. While the complexity of such mechanisms do highlight overlapping roles and effects, such key regulatory roles do have great significance in being considered further, particularly if exploited as potential targets for GC therapy in a Correa stage-specific model for GC development and progression (Figs. 1, 4).

## Current treatments for *H. pylori*-mediated gastric cancer

Observational studies have been conclusive in linking *H. pylori* infection with increased GC risk [14], as seen with the eradication of *H. pylori* infection decreasing such a risk by approximately 40% in asymptomatic patients and recurrence by 54% in individuals who had undergone endoscopic resection for GC [212, 213]. Currently, the recognized critical point, beyond which the eradication of *H. pylori* does not prevent the progression of GC, is between the Correa model stages of metaplasia and dysplasia. While in some studies where GC had a high prevalence (such as in SE Asia), a risk reduction was observed through *H. pylori* eradication programs [213], uncertainty remains as to whether such findings can be extrapolated to countries where there is a low prevalence. Available therapeutic regimes for GC are very diverse and can take a number of forms based on the stage of development. For example, the use of proton pump inhibitors (PPIs) are universally administered for the treatment of *H. pylori* infection during chronic gastritis [214], and their efficacy can be improved when offered in a combined therapy along with antibiotics, such as clarithromycin, amoxicillin and metronidazole [215].

In addition to the Correa model for GC, histological Borrmann classifications based upon macroscopic appearance [216] can also allow patients to be sub-grouped, followed by staging, surgical and endoscopic resection or chemotherapy. Of importance, chemotherapy before curative resection can also offer enhanced 5-year survival rates and cure rates of up to 40%, and is the recommended standard treatment for patients with locally advanced GC [217–219]. The emerging use of antibody therapeutics has offered encouragement, based on them being able to activate host immunity in cancer cell recognition and clearance [220], as have the alternative uses of cytokine-induced killer (CIK) cell therapy [221]. Here, immune checkpoint antibody inhibitors that can block immunosuppressive signals are offering a promising approach. For example, the use of pembrolizumub has showed an encouraging 6-month progression-free survival of 24% and an overall survival (OS) of 33%, in treating late-stage GC patients [222]. As a more recent example, trastuzumab deruxtecan is also showing encouraging outcomes for the treatment of HER2-positive pre-treated GC patients following phase I/II clinical trials [223, 224]. As a next-generation antibody therapeutic, it is composed of an anti-Her2 antibody fused to the topoisomerase I inhibitor DXd, through a cleavable linker and showed enhanced objective response rates and overall survival of patients who had previously received at least two lines of chemotherapy [225]. Additionally, trastuzumab combined with fluoropyrimidine and platinum chemotherapy has also been seen to prolong the OS of patients with HER-2-positive inoperable GCs [226]. Other excellent chemotherapeutics that also show great potential for such uses include platinum-fluoropyrimidene [227–229], cisplatin, oxaliplatin, irinotecan and fluoropyrimidines [230]. Moreover, cell cycle inhibitors such as flavopiridol have also gained some attention recently, based on their promising efficacy as small molecule inhibitors that can target the cyclin-dependent kinases [231], and are currently being administered as combined therapeutics with docetaxel in GC therapy [232]. Finally, how such therapeutic regimens modulate cathepsin protease activity or expression is also an area of significance, based on the input these proteases have on regulation of inflammation or cancer progression, and therefore warrant further evaluation in such studies (Table 4).

**Table 4:** Selective antibody therapeutics currently being trialed in combination or as pre-treatments with conventional chemotherapeutics

Therapeutic (disease)	Combined therapeutic	GC target (cell type)		Target effects in GC	Cathepsin expression	References
Ramucirumab (GC)	Paclitaxel	VEGFR-2 (tumor)		Angiogenesis, metastasis	Uk	[233]
Sorafenib (GC)	Cisplatin Docetaxel	VEGFR1-3 (tumor)		Angiogenesis, metastasis	Cathepsin B (+)	[234] [235]
Bevacizumab (RP/GC)	Irinotecan Cisplatin	VEGF-A-L (tumor)		Angiogenesis, metastasis	Uk	[236]
					Cathepsin D (+)	[237]
Centuximab (GC)	Cisplatin	EGF-R (tumor)		Proliferation, migration, Ang	Uk	[238]
Panitumumab (OS/GC)	Docetaxel Cisplatin	EGF-R (tumor)		Proliferation, migration, Ang	Uk	[239]
					Uk	[240]
Nimotuzumab (GC)	Cisplatin Docetaxel/Cisplatin	EGF-R (tumor)		Proliferation, migration, Ang	Uk	[241]
					Uk	[242]
Avelumab (CRC)	–	PD-1L (tumor)		Imm. Surv TME,TCF	Uk	[243, 244]
Pembrolizumab (CRC) Nivolumab (GC)	– Pre-treatment	PD-1 (T cell)		Imm. Surv TME, TCF	Uk	[222]
					Uk	[245]
Trastuzumab deruxtecan (GC)	Anti-PD-1	EGF-R2 (tumor)		Imm. Surv migration	Uk	[246]

Target proteins and their effects on gastric cancer progression or cathepsin protease expression are highlighted. *GC* gastric cancer, *CRC* colorectal cancer, *RP* retinal pigment cells, *OS* esophagogastric cancer, *VEGF* vascular endothelial growth factor, *Imm. Surv.* immunosurveillance, *TME* tumor microenvironment, *Ang.* angiogenesis, *TCF* T-cell function; induced (+); decreased (−); unchanged (u); unknown (Uk)

To summarize, the treatments offered for GC development beyond the *H. pylori* infection stage generally offer limited curative success or OS of patients up to 5 years or beyond, and while combined strategies show some encouraging outcomes, alternative strategies do need to be given greater consideration, for which the input of the intracellular or extracellular cathepsin proteases cannot be ignored.

## Conclusions

In conclusion, the effects that microbes can have in relation to disease onset are very real and in some instances are also being unveiled as an integral part of disease progression. In the context of *H. pylori*, its role in GC onset and progression is relatively asymptomatic, but over time can give rise to tangible effects with life-threatening consequences. While the initial inert effects of *H. pylori* infection can be addressed in the clinic with relative ease, its long-term effects culminating through chronic inflammation to GC progression offer little in the way of effective therapy outside of surgical resection. Throughout GC progression, a whole array of significant biological and physiological effects can take place, which offer the basic research communities a number of significant areas for exploration, with a view to mechanistically defining with greater clarity the regulation of the immune response and how it appears to be ‘annexed’ by developing tumors to aid their own progression. While not all types of cancers may share such an etiological component, the growing recognition of the immune response and its involvement in cancer development are nevertheless firmly established. At the cellular level, the input of neutrophils, dendritic cells, macrophages, mast cells and cells from the adaptive immune system share a very complex relationship and, through sheer resilience, the scientific community is unravelling the relationships such cellular components share with each other or with the tumor, and particularly in the context of GC progression. At the heart of this is the dynamic relationship shared by the immune system, the ECM and the TME in permitting a whole raft of effects to take place in permitting the growth of tumor cells, their metastasis and vascularization. Indeed, key proteins that help in modulating such a dynamic at the molecular level, which are being viewed to carry significance throughout disease progression, are the cathepsin proteases. While originally thought to be just lysosomal proteases, essential to the process of protein degradation, they are without question emerging as essential players during microbial-mediated disease progression, through their diverse activities extracellularly and within the cell.

## Data Availability

All data pertaining to this manuscript is available for scrutiny upon request.

## Abbreviations

APC: Antigen-presenting cells
BMDC: Bone marrow-derived cells
BMDSC: Bone marrow-derived stem/suppressor cells
CAF: Cancer-associated fibroblast cells
CagA: Cytotoxicity-associated gene A protein
CTLS: Cytotoxic T cells
ECM: Extracellular matrix
EMT: Epithelial–mesenchymal transition
GC: Gastric cancer
MALT: Mucosa-associated lymphoid tissue
MAPK: Mitogen-activated protein kinases
MMP: Matrix metalloprotease proteins
MOMP: Mitochondrial outer membrane permeabilization
NK: Natural killer cells
PAMP: Pathogen-associated molecular pattern proteins
ROS: Reactive oxygen species
RNS: Reactive nitrogenous species
TAF: Tumor-associated fibroblast cells
TAM: Tumor-associated macrophage cells
TIN: Tumor-infiltrating neutrophil cells
TLR: Toll-like receptor proteins
TME: Tumor microenvironment
VacA: Vacuolating cytotoxic A protein
WHO: World Health Organization
